# Targeting the Interleukin-23/Interleukin-17 Inflammatory Pathway: Successes and Failures in the Treatment of Axial Spondyloarthritis

**DOI:** 10.3389/fimmu.2021.715510

**Published:** 2021-09-03

**Authors:** Runsheng Wang, Walter P. Maksymowych

**Affiliations:** ^1^Division of Rheumatology, Columbia University Irving Medical Center, New York, NY, United States; ^2^Garden State Rheumatology Consultants, Union, NJ, United States; ^3^Department of Medicine, University of Alberta, Edmonton, AB, Canada; ^4^CARE Arthritis, Edmonton, AB, Canada

**Keywords:** axial spondylarthritis, treatment, IL-23/IL-17 axis, inflammation, disease progression

## Abstract

The IL-23/IL-17 pathway has been implicated in the etiopathogenesis of axial spondyloarthritis through studies of genetic polymorphisms associated with disease, an animal model with over-expression of IL-23 that resembles human disease, and observations that cytokines in this pathway can be found at the site of disease in both humans and animal models. However, the most direct evidence has emerged from clinical trials of agents targeting cytokines in this pathway. Monoclonal antibodies targeting IL-17A have been shown to ameliorate signs and symptoms, as well as MRI inflammation in the spine and sacroiliac joints, in patients with radiographic and non-radiographic axial spondyloarthritis. This was evident in patients refractory to non-steroidal anti-inflammatory agents as well as patients failing treatment with tumor necrosis factor inhibitor therapies. Treatment with a bispecific antibody targeting both IL-17A and IL-17F was also effective in a phase II study. Post-hoc analyses have even suggested a potential disease-modifying effect in reducing development of spinal ankylosis. However, benefits for extra-articular manifestations were limited to psoriasis and did not extend to colitis and uveitis. Conversely, trials of therapies targeting IL-23 did not demonstrate any significant impact on signs, symptoms, and MRI inflammation in axial spondyloarthritis. These developments coincide with recent observations that expression of these cytokines is evident in many different cell types with roles in innate as well as adaptive immunity. Moreover, evidence has emerged for the existence of both IL-23-dependent and IL-23-independent pathways regulating expression of IL-17, potentially associated with different roles in intestinal and axial skeletal inflammation.

## Introduction

Axial spondyloarthritis (axSpA) is an inflammatory disease of the sacroiliac joints (SIJ) and spine that may also involve peripheral joints and entheses (1). It is associated with extra-articular sites of inflammation manifesting as anterior uveitis, aortitis, colitis, and psoriasis. A pathological hallmark is the development of new bone formation as a tissue response to inflammation which is primarily evident in the SIJ and spine. This may lead to spinal ankylosis, and its severity determines the degree of functional impairment. Disease onset is typically in the third and fourth decades of life and the disease pursues a severe course in about 30-40% of cases, with considerably impairment of quality of life and even increased mortality (2). There is a strong genetic component to its etiology along with alterations in gut microbiome and mechanical factors though the precise steps remain unclear (3). Therapeutics that effectively control inflammation have recently emerged, although these have been identified more by randomized placebo-controlled trials (RCTs) than by studies of basic pathogenesis. Therapeutic agents that target tumor necrosis factor (TNF) have now been used to successfully treat not only joint and entheseal inflammation but also all extra-articular manifestations of disease (4, 5). It remains unclear, however, whether they impact the development of new bone, and about 30-40% of patients fail to attain a sufficient response. Recent attention has pivoted to the interleukin-23(IL-23)/interleukin-17(IL-17) pathway as data has emerged from animal models and human tissue samples that these cytokines are present at the site of disease and are key effectors of tissue inflammation. However, the picture that has emerged is a complex one as it has become apparent that targeting this pathway is effective in some tissue locations and not others. Our aims in this review were to discuss recent studies investigating the role of the IL-23/IL-17 pathway in the pathophysiology of inflammation and new bone formation in axSpA, data from recent RCTs and other studies informing the tissue specificity of IL-23 and IL-17 targeted therapies, and the new implications of the findings of RCTs for our understanding of the pathophysiology of axSpA.

## Classic IL-23/IL-17 Pathway

**Figure 1:**
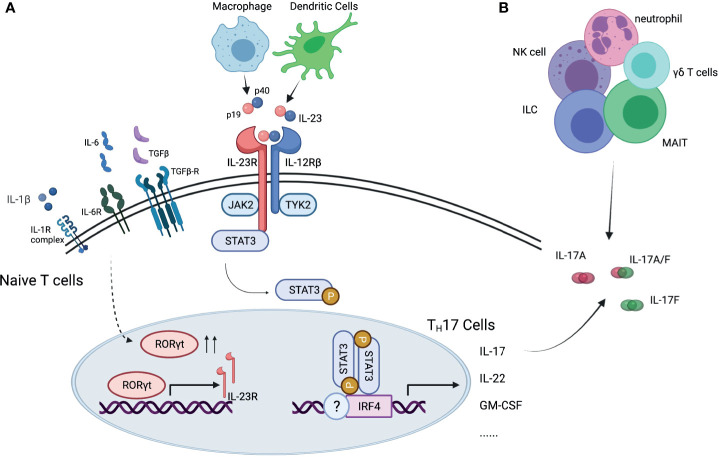
Interleukin-23/interleukin-17 pathway. **(A)** IL-23 is essential for differentiation of T_H_17 cells and production of IL-17 cytokine. **(B)** IL-17 cytokine can be produced by other immune cells. IL, interleukin; IL-23R, interleukin-23 receptor; IL-12Rβ, interleukin-12 receptor beta; IL-6R, interleukin-6 receptor; TGFβ, transforming growth factor beta; TGFβ-R, transforming growth factor beta receptor; IL-1R, interleukin-1 receptor; JAK2, Janus kinase 2; TYK2, tyrosine kinase 2; RORγt, retinoid-related orphan receptor gamma t; receptor- STAT3, signal transducer and activator of transcription 3; IRF4, interferon regulatory factor 4; NK cell, natural killer cell; ILC, innate lymphoid cell; MAIT, mucosal associated invariant T cells.

IL-23 is a pro-inflammatory cytokine with a critical role in mediating autoimmunity (**Figure 1**). It promotes the development of a group of cells that express transcriptional factor retinoid-acid-receptor-related orphan receptor γ (RORγt), including T_H_17 cells differentiated from naïve T cells, and other IL-17 expressing cells in the innate immune system or non-lymphoid tissues. These RORγt+ cells express a unique panel of inflammatory cytokines and chemokines, including IL-17, IL-22 and granulocyte-macrophage colony-stimulating factor (GM-CSF) (6, 7). The recognition of IL-23 and downstream T_H_17 cells challenged the long-standing paradigm of T_H_1- T_H_2 cells, adding T_H_17 cells in the spectrum of immune response (8).

IL-23 is a heterodimer with two subunits, the p40 subunit, shared with IL-12, and the p19 subunit, unique to IL-23. It is primarily secreted by macrophages and dendritic cells in tissues like skin, intestinal mucosa, lungs, synovium, and brain, and signals through the IL-23 receptor (IL-23R) complex. The IL-23R complex consists of two subunits, one is IL23R, and the other is IL-12 receptor (IL-12R) β chain (9), shared with IL-12R. IL-23/IL-23R signaling plays an important role in T_H_17cell differentiation, survival, and expansion. The initial stage of T_H_17 cell differentiation from naïve T cells is triggered by IL-6, transforming growth factor β (TGFβ), and IL-1β. The presence of these cytokines promotes the expression of RORγt, signal transducer and activator of transcription 3 (STAT3) and interferon-regulatory factor 4 (IRF4). After the initial trigger, IL-23 is essential for maturation of T_H_17 cells, by reinforcing RORγt expression and suppresses differentiation to T_H_1, T_H_2 or regulatory T cells (Treg) (10), as well as for maintenance of T_H_17 signature gene expression, including IL-17, IL-22 and GM-CSF (11).

**Figure 2:**
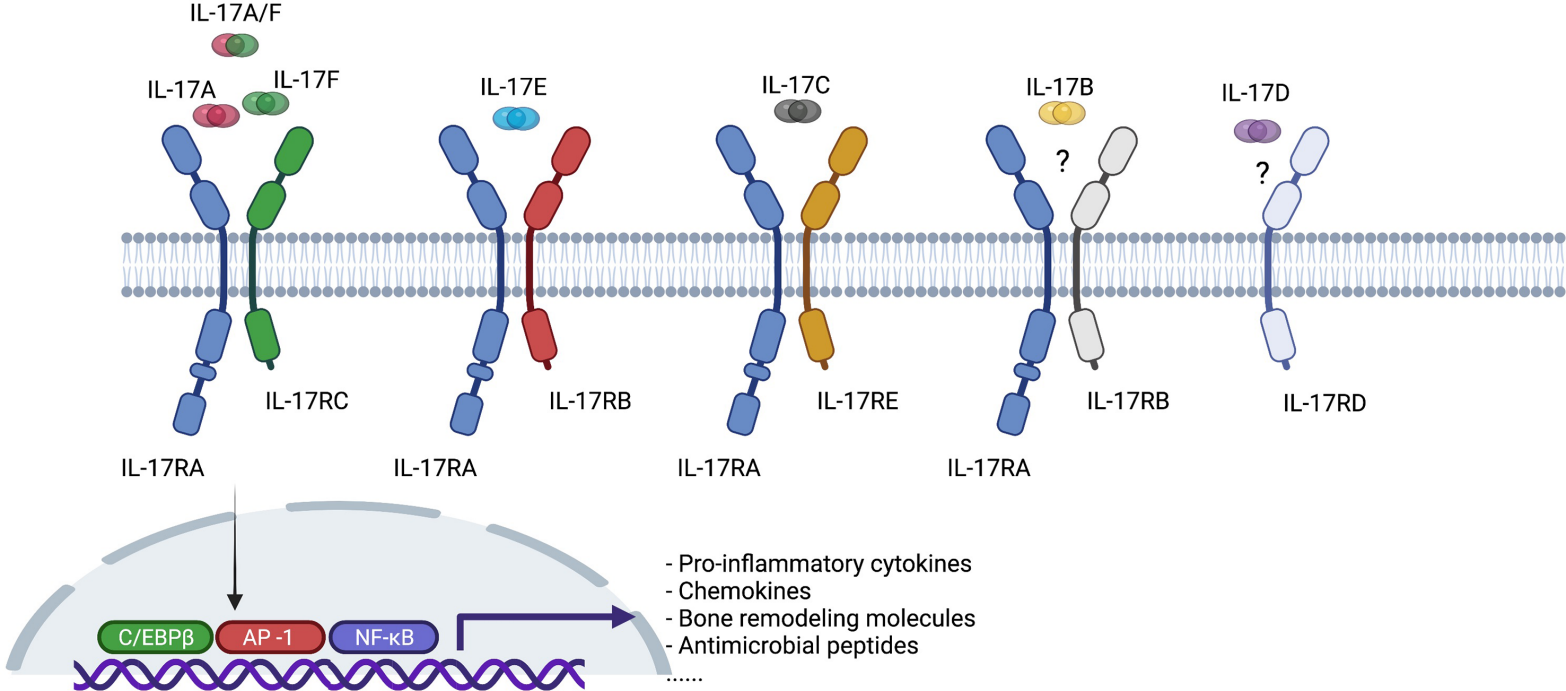
Interleukin-17 (IL-17) cytokine family and interleukin-17 receptor (IL-17R) family. C/EBPβ, CCAAT-enhancer-binding protein beta; AP-1, activator protein-1; NK-κB, nuclear factor kappa B.

IL-17 is a family of pro-inflammatory cytokines primarily secreted by T_H_17 cells, and by natural killer cells (NK cells), innate lymphoid cells (ILCs), γδ T cells, mucosal-associated invariant T (MAIT) cells. It has six members, from IL-17A to IL-17F, IL-17A being the best described among them (**Figure 2**) (11, 12). It binds to IL-17 receptors, a family of receptors that consist of 5 homologous subunits, IL-17RA to IL17-RE, that are widely expressed in all type of cells. Among them, IL-17RA is the most well studied, primarily expressed in myeloid cells, while IL-17RC is mainly expressed in non-hematopoietic cells, such as epithelial cells, mesenchymal cells and fibroblasts. IL-17 induces expression of pro-inflammatory cytokines (e.g. IL-6 and IL-8) and chemokines that promote neutrophil chemotaxis and accumulation, antimicrobial peptides that protect skin and mucosal surfaces, and molecules that are involved in bone remodeling [e.g. matrix metalloproteinases (MMPs) and receptor activator for nuclear factor-κB ligand (RANKL)] (13, 14).

IL-17A and IL-17F are typically co-expressed by type 17 cells and exist as both homodimers and as a heterodimer. All forms bind to a heterodimeric IL-17RA/IL-17RC complex and trigger qualitatively similar signaling pathways. Both cytokines are increased in a variety of inflamed human tissues and there is synergistic activity with other proinflammatory cytokines, such as TNF, in driving inflammatory pathways (15–17). In a proof-of-concept study of skin and synovial tissue from patients with psoriatic arthritis (PsA), both IL-17A and IL-17F were present in skin lesions and inflamed synovial tissue (18). Moreover, IL-17A or IL-17F did not activate PsA synoviocytes when administered alone, but both cytokines stimulated PsA synoviocytes to produce pro-inflammatory cytokines such as IL-6 and IL-8 when co-administered with TNF, although IL-17F was less potent than IL-17A. Dual neutralization of IL-17A and IL-17F demonstrated greater suppression of synoviocyte and healthy human dermal fibroblast activation and decreased expression of IL-6, IL-8, and metalloproteinase-3 (MMP3) than blockade of IL-17A or IL-17F alone. Of particular relevance to axSpA, IL-17F mediates osteogenic differentiation of human periosteal cells induced by supernatant from T_H_17 or gamma-delta T cells and this can be inhibited using an antibody targeting IL-17F (19). Moreover, a bispecific antibody neutralizing both cytokines, bimekizumab, was more effective than antibodies targeting either IL-17A or IL-17F implying that such dual inhibition might prevent the ankylosis observed in patients with axSpA.

Relatively little is known about the pathophysiological role of other IL-17 family members relevant to axSpA. IL-17B may have an anti-inflammatory role by blocking IL-17E signaling during mucosal inflammation (20). IL-17C may have a similar role to IL-17A in anti-microbial protective responses in the intestine but is produced by epithelial rather than immune cells and may therefore provide a rapid local response to epithelial injury (21). A recent report indicated that IL-17D is the most highly expressed IL-17 family member in inflamed SpA synovium and is expressed by synovial stromal cells (22). The level of expression of IL-17D correlated with levels of expression of genes associated with synovial tissue remodeling (e.g. Bone Morphogenetic Protein (BMP) 4, BMP Receptor Type 1B, TGFβ Receptor 3, Wnt Family Members 3 and 11, Fibroblast Growth Factor 1 and 17) and varied inversely with the degree of expression of genes associated with inflammation. IL-17D deficiency in mice increases joint and systemic inflammation suggesting that the cytokine has an anti-inflammatory role in joint inflammation. IL-17E is expressed by intestinal tuft cells and has a beneficial role in mucosal immunity to parasitic infection but has also been shown to inhibit autoimmunity induced by T_H_17 cells and suppress production of IL-1, IL-6, and IL-23 by activated dendritic cells (23).

Case reports of patients with monogenic mutations in the IL-23/IL-17 pathway indicate that, physiologically, IL-23 and IL-17 are involved in maintaining mucosa barrier and protecting the body against bacterial and fungal infections. Increased risk for mucocutaneous candidiasis has been reported in patients with loss of function in IL-17F, IL-17RA or IL-17RC (24, 25). Patients with autoimmune polyglandular syndrome type 1 (APS-1) syndrome have neutralizing antibodies against IL-17A, IL-17F and/or IL-22, and have increased risk for candidiasis (26). Patients with autosomal dominant hyper-IgE syndrome (Job’s syndrome, caused by *STAT3* mutation) have defects in IL-6, IL-23 and IL-22 signaling with reduced T_H_17 cells, and are prone to have mucocutaneous candidiasis, staphylococcus aureus infection, and probably viral infections (27).

### IL-17/IL-23 Pathway in Axial Spondyloarthritis

Human genome wide association analyses (GWAS) and studies in animal models and human tissues have implicated a pivotal role of IL-23/IL-17 pathway in the disease pathogenesis of ankylosing spondylitis (AS, a.k.a. radiographic axSpA, r-axSpA).

GWAS showed that *IL23R* (rs11209026, rs1004819, rs10489629, rs11465804, rs1343151, rs10889677, rs11209032, rs1495965) and *IL12B* (rs6556416, rs10045431) single nuclear polymorphism (SNPs) are associated with the susceptibility to AS, as well as SpA related conditions, such as psoriasis and inflammatory bowel disease (IBD) (28–30). In addition, GWAS of Vogt-Koyanagi-Harada (VKH) syndrome, a condition that primarily manifests as pan-uveitis, also showed that the *IL23R* SNP is associated with increased disease susceptibility (31). Apart from these two genes, genes that modulate the IL-23/IL-17 pathway, such as *STAT3* (rs6503695, rs744166), *IL1R2* (rs2310173), *CARD9* (rs10781500), have been reported to be associated with the risk of having AS and IBD (32). The SNP of *TNFAIP3*, the gene that encodes A20 protein and inhibits IL-17 signaling (33), was associated with risk for psoriasis (30).

Consistent with the genetic findings, *in vivo* over-expression of IL-23 in mouse models induces enthesitis, the pathologic hallmark of axSpA. In B10.RIII mice, IL-23 overexpression acts on a group of ROR-γt+ entheseal resident CD4 and CD8 negative T cells, and induces expression of IL-17 and IL-22, as well as IL-6 and Chemokine Ligand 1 (34). Additional features resembling the human axSpA phenotype included psoriasiform skin lesions, aortitis, uveitis, peripheral arthritis, and spondylitis. A subsequent report demonstrated that γδ T cells are the major cells producing IL17 in the enthesis of this IL-23 overexpressing model and that 50-80% of these cells are of the Vγ6+ phenotype (35). Furthermore, these cells also accumulate in the aortic valve and root as well as the ciliary body of the eye. However, this model has proven difficult to reproduce in other labs. In one report, over-expression of IL-23 using minicircle DNA led to chronic arthritis, severe bone loss, and myelopoiesis associated with the expansion of a myeloid lineage osteoclast precursor. This was partly dependent on TNF and IL-17A but could not be reproduced by overexpression of IL-17A (36). In SKG mice, after injection of curdlan, the mice developed IL-23 and T cell dependent arthritis and spondylitis, and the phenotype was transferrable by CD4+ T cells (37). Interestingly, a study in the HLA-B27 transgenic rat model with arthritis and spondylitis showed that IL-23R inhibition is effective for disease prevention when given prior to clinical onset, but when used for treatment of established disease, anti-IL-23R did not reduce clinical features or levels of IL-17 and IL-22 (38). Instead, when treated with anti-IL17A, the axial and peripheral joint inflammation were significantly reduced (39), suggesting IL-23 might be responsible for the onset of axSpA, but not for maintenance of the phenotype.

Immunohistochemical analysis of facet joints from patients with AS confirmed the presence of IL-23 expressing cells, including myeloperoxidase positive cells, macrophages and dendritic cells (40), as well as IL-17 positive neutrophils, T cells and mast cells (41). In peripheral blood of patients with AS, an increase of IL-23R expressing γδ T cells was also observed, associated with enhanced IL-17 secretion from these cells (42).

In summary, preclinical studies provided indirect evidence for inhibiting IL-23 or IL-17 as potential treatment options for axSpA.

## Clinical Studies of IL-17 Inhibition in Axial Spondyloarthritis

Monoclonal antibodies against IL-17A alone or with dual inhibition of IL-17A/IL-17F have been developed and tested in clinical trials of axSpA, with clear efficacy in treating clinical symptoms and reducing inflammation.

### Secukinumab

The first approved IL-17A antibody for AS was secukinumab (SEC), a fully human IgG1κ monoclonal antibody. MEASURE 1 and MEASURE 2 were two randomized, double-blind, placebo controlled, phase 3 trials in patients with active AS, defined by the modified New York criteria (see **Box 1**) (43), with prior exposure to no more than one tumor necrosis factor inhibitor (TNFi). Patients were randomized 1:1:1 into placebo, SEC 150mg every 4 weeks (Q4W), and SEC 75mg Q4W arms, with either intravenous loading dose in MEASURE 1 or subcutaneous loading dose in MEASURE 2. The primary endpoint was the Assessment of Spondyloarthritis International Society 20% response criterion [ASAS20 (44), see **Box 1**] at Week 16. Secondary endpoints included the ASAS40 response (44) (see **Box 1**), change of Bath AS disease activity index [BASDAI (45), see **Box 1**], SF-36, AS Quality of Life (ASQoL) scale, and ASAS partial remission. In MEASURE 1, 371 patients were randomized, with 61% of patients in the SEC 150mg arm, 60% of patients in the SEC 75mg arm *versus* 29% of patients in the placebo arm meeting the primary endpoint. ASAS40 responses were 42%, 33% and 13% in these three arms, respectively. In MEASURE 2, 219 patients were randomized, and 61% of patients in the SEC 150mg arm, 41% of patients in the SEC 75mg arm, *versus* 28% of patients in the placebo arm met the primary endpoint. ASAS40 responses were 36%, 26% and 11% in these three arms, respectively (51) (**Table 1**). All other secondary endpoints were met, except ASAS partial remission in the MEASURE 2 study. Long term extension studies of SEC up to 5 years follow up showed that, for those who stayed on SEC for two years, 84% patients (N = 230/274) remained on SEC at Year 5, with an ASAS40 response rate of 65.2% and BASDAI of 2.6, indicating a very low level of active symptomatology (52).

**Table 1:** Pivotal randomized clinical trials of IL-17 inhibitors in axial spondyloarthritis.

Study ID	Drug	Acronym	Clinical Trial Phase	Condition	Previous bDMARD use	Primary Endpoint	TNF inaïve (%)	Arms	Enrollment (n)	Meeting Primary Endpoint
NCT01358175	SEC	MEASURE 1	Ph. 3	AS	Not more than one TNFi	W16 ASAS20	73%	Placebo	122	29%
							73%	SEC IV loading + 150mg Q4W	125	61%
							74%	SEC IV loading + 75mg Q4W	124	60%
NCT01649375	SEC	MEASURE 2	Ph. 3	AS	Not more than one TNFi	W16 ASAS20	61%	Placebo	74	28%
							61%	SEC IV loading + 150mg Q4W	72	61%
							62%	SEC IV loading + 75mg Q4W	73	41%
NCT02696031	SEC	PREVENT	Ph. 3	nr-axSpA	Prior TNFi allowed	W16 ASAS40*	91.9%	Placebo	186	29.2%
							88.6%	SEC LD + 150mg Q4W	185	41.5%
							90.2%	SEC NL + 150mg Q4L	184	42.2%
NCT02696798	SEC	COAST-V	Ph. 3	AS	Not allowed	W16 ASAS40	100%	Placebo	87	18%
							100%	ADA 40mg Q2W	90	32%
							100%	IXE 80mg Q2W	83	52%
							100%	IXE 80mg Q4W	81	48%
NCT02757352	IXE	COAST-W	Ph. 3	r-axSpA	TNFi experienced required	W16 ASAS40	0%	Placebo	104	12.5%
							0%	IXE 80mg Q2W	98	30.6%
							0%	IXE 80mg Q4W	114	25.4%
NCT02696785	IXE	COAST-X	Ph. 3	nr-axSpA	Not allowed	W16 ASAS40*	100%	Placebo	105	19%
							100%	IXE 80mg Q2W	102	41%
							100%	IXE 80mg Q4W	96	35%
NCT02963506	BKZ	BE AGILE	Ph. 2B	AS	TNFi experienced allowed	W12 ASAS40	88.3%	Placebo	60	13.3%
							86.9%	BKZ 16mg Q4W	61	29.5%
							88.5%	BKZ 64mg Q4W	60	42.6%
							88.3%	BKZ 160mg Q4W	60	46.7%
							91.8%	BKZ 320mg Q4W	61	45.9%
NCT02763111	NTK	–	Ph. 2	AS	TNFi experienced allowed	W16 ASAS20	81.8%	Placebo	22	42.9%
							81.8%	NTK 40mg	22	72.7%
							90.9%	NTK 80mg	22	81.8%
							86.4%	NTK 120mg	22	90.9%

SEC, secukinumab; IXE, ixekizumab; BKZ, bimekizumab; NTK, netakimab; AS, ankylosing Spondylitis; nr-axSpA, nonradiographic axial spondyloarthritis; r-axSpA, radiographic axial spondyloarthritis; bDMARD, biologic disease modifying anti-rheumatic drug; TNFi, tumor necrosis factor inhibitor; ASAS20, the Assessment in Ankylosing Spondylitis 20% response criteria; ASAS40, the Assessment in Ankylosing Spondylitis 40% response criteria.

**Box 1 | Fact Box. Diagnoses and major outcome measures in axial spondyloarthritis.**

Ankylosing spondylitis (AS) – classified based on modified New York (mNY) criteria, composed of clinical criteria (inflammatory back pain (IBP), limited range of motion in lumbar spine, or limited range of motion in chest expansion) and radiological criterion (bilateral grade 2 sacroiliitis or unilateral grade 3 sacroiliitis on radiograph) (43). Definite AS is defined as meeting radiologic criterion and at least one clinical criterion.

Axial Spondyloarthritis (axSpA) – classified based on Assessment of SpondyloArthritis international Society (ASAS) criteria (44). In patients with 3 months back pain and age of onset < 45 years, axial spondyloarthritis is defined as 1) having sacroiliitis on imaging studies (either radiographic according to mNY criteria or magnetic resonance imaging (MRI) evidence of subchondral bone marrow edema highly suggestive of axSpA) and one spondyloarthritis (SpA) feature (IBP, arthritis, heel enthesitis, uveitis, dactylitis, psoriasis, Crohn’s/colitis, good response to non-steroidal anti-inflammatory drugs, family history of SpA, positive HLA-B27, elevated C-reactive protein), or 2) positive HLA-B27 and two other SpA features. The condition is further categorized as radiographic axSpA (r-axSpA) if sacroiliitis is present on pelvis radiograph, and non-radiographic axSpA (nr-axSpA) if not present.

ASAS 40% response criteria (ASAS40) – improvement of ≥ 40% and ≥2 units (0-10 scale) in at least three of the four domains (patient global, pain, function, inflammation), and no worsening in any domain (44).

Bath ankylosing spondylitis disease activity index (BASDAI) – a six-question (each with 0-10 scale), self-administered questionnaire, assessing fatigue, spinal pain, peripheral arthritis, enthesitis, intensity and duration of morning stiffness in patients with AS (45).

Bath ankylosing spondylitis functional index (BASFI) - a 10-question (each with 0-10 scale), self-administered questionnaire, assessing degree of functional limitations in patients with AS (46).

Ankylosing spondylitis disease activity score (ASDAS) - a composite score, including assessment of total back pain, patient global of disease activity, peripheral pain and swelling, duration of morning stiffness, and C-reactive protein or erythrocyte sedimentation rate (47).

Spondyloarthritis Research Consortium of Canada MRI index for scoring inflammation in the sacroiliac joints (SIJ) (SPARCC MRI SIJ score) - a semi-quantitative scoring system to assess active inflammation in SIJs, based on the presence, intensity and depth of bone marrow edema on a fat-suppressed sequence in six consecutive coronal slices of pelvis MRI, with a total maximum score of 72 (48).

Spondyloarthritis Research Consortium of Canada MRI index for scoring inflammation in the spine (SPARCC MRI Spine score) - a semi-quantitative scoring system to assess active inflammation in the spine, based on the presence, intensity and depth of bone marrow edema on a fat-suppressed sequence of 3 consecutive sagittal slices through each discovertebral level of the entire spine, with a total maximum score of 414 (49). Modified Stoke AS Spine Score (mSASSS) - a semi-quantitative scoring system to assess structural damage of the spine based on certain features (erosion, sclerosis, squaring, syndesmophytes and bony bridging between adjacent vertebra) of the anterior vertebral corners on lateral projections of cervical and lumbar spine radiographs, with a total maximum score of 0 -72 (50).

A 1-year placebo-controlled RCT of SEC recruited 555 patients with non-radiographic axial SpA (nr-axSpA) who were randomized (1:1:1) to receive subcutaneous SEC 150 mg with a loading dose (loading dose [LD] group), SEC 150 mg without a loading dose (non–loading dose [NL] group), or placebo weekly and then Q4W starting at week 4 (53). The primary endpoint was the ASAS40 response at week 16 (European Union and non-US analysis) and week 52 (US analysis). Escape to open label SEC was possible at any time after week 20. Most recruited patients were naïve to TNFi (90.3%) and in these patients an ASAS40 response was achieved at 16 weeks in 41.5% of the SEC 150mg LD group, 42.2% of the SEC NL group, and 29.2% of those on placebo (**Table 1**). The corresponding response rates at week 52 were 35.4%, 39.8%, and 19.9% of patients in the SEC 150mg LD, SEC 150mg NL, and placebo groups, respectively. All major secondary endpoints showed greater improvement in the SEC groups compared to those on placebo at week 16. These included the ASAS5/6 response, ASAS partial remission, BASDAI50 response at weeks 16, change from baseline to week 16 in the BASDAI, high sensitivity C-reactive protein (CRP), Bath AS Functional Index [BASFI (46), see **Box 1**], MRI SIJ edema (Berlin score), SF-36 Physical Component Scale, and ASQoL score. For the week 52 analysis of secondary endpoints, significance *versus* placebo was achieved for the BASDAI50 and AS Disease Activity Score [ASDAS (47), see **Box 1**] inactive disease [(SDAS-ID, defined as an ASDAS score <1.3) responses in the SEC 150mg NL group but not for ASDAS-ID in the SEC LD group and in view of the hierarchical testing procedure SIJ edema score on MRI and ASQoL for the 150 mg LD group were not tested. The frequencies of serious adverse events (SAE) and discontinuations due to adverse events were similar across the three groups. There were 14 cases of uveitis in 11 patients, 9 in the SEC groups (4 *de novo* cases) and 2 in the placebo group. Seven patients receiving SEC reported colitis (5 Crohn’s disease and 2 ulcerative colitis) of whom two had a history of colitis.

Overall, the SEC RCT data demonstrates convincingly that IL-17A targeted therapy with this agent is effective at ameliorating inflammation in AS as well as nr-axSpA patients who are biologic disease modifying anti-rheumatic drug (bDMARD) naïve and refractory to non-steroidal anti-inflammatory drug (NSAIDs) therapy, but the trials recruited insufficient numbers of TNFi experienced patients. Moreover, a trial recruiting only TNFi experienced patients has not yet been reported. The impact on concomitant musculoskeletal inflammation in the peripheral skeleton in axSpA, such as enthesitis and peripheral synovitis, has also not yet been reported.

### Ixekizumab

Ixekizumab (IXE) is another IL-17A inhibitor that have been approved in treating active axSpA. It is a humanized IgG4 monoclonal antibody. In the COAST-V study, a phase 3, double-blinded RCT, biologic naïve patients with active r-axSpA based on ASAS classification criteria (44) (see **Box 1**) were included in the study (54). In addition to the placebo control, the study also had an active comparator arm of adalimumab 40mg Q4W, a fully human IgG1 monoclonal antibody to TNFα. The treatment arms included IXE 80mg every 2 weeks (Q2W) and IXE 80mg Q4W. The primary endpoint of the study was the proportion of patients who achieved an ASAS40 response at Week 16. The main secondary endpoints included the ASAS20 response, the BASDAI50 response, change in ASDAS, change in BASFI, and ASDAS-ID. In this study, MRI of spine and SIJ were obtained at baseline and Week 16, and change in the severity of MRI inflammation in the spine was assessed using the Spondyloarthritis Research Consortium of Canada (SPARCC) MRI spine score (see **Box 1**)while change in the severity of MRI inflammation in the sacroiliac joints was assessed using the SPARCC MRI SIJ score (see **Box 1**) and these were among the secondary outcomes (48, 49). A total of 341 patients were included and randomized 1:1:1:1 into 4 arms. At Week 16, significantly more patients in the IXE Q2W arm (51.8%), the IXE Q4W arm (48.1%), and the adalimumab arm (35.6%) achieved an ASAS40 response, compared to patients in the placebo arm (18.4%) (**Table 1**). The main secondary outcomes were significantly better in the active treatment compared to placebo arms, including the SPARCC MRI spine and SIJ scores. During the 16 weeks of the double blinded period, the most common AEs were nasopharyngitis, which occurred in 23 patients, and almost equally among the 4 arms (6.0% to 7.4%). One patient in each IXE arm, and 3 in the adalimumab arm experienced SAE. Three patients in the IXE Q2W arm and one in the adalimumab arm discontinued the study due to AEs. One case of candida infection in the adalimumab arm was reported, as well as one case of cerebrocardiovascular event in the IXE Q4W arm, one case of IBD in the IXE Q2W arm, and one case of depression in the adalimumab arm.

The COAST-W study of IXE followed a similar design to COAST-V, except that patients in COAST-W were required to have discontinued at least one TNFi, but not more than 2 TNFi, due to inadequate response or intolerance (55). A total of 316 patients were included and randomized 1:1:1 into placebo, IXE 80mg Q2W and IXE 80mg Q4W arms. At Week 16, significantly more patients in the IXE Q2W arm (30.6%) and the IXE Q4W arm (25.4%) achieved ASAS40, compared to placebo (12.5%) (**Table 1**). A proportion of patients had spine MRI at baseline and Week 16. The reductions in the MRI SPARCC spine scores were significantly more in the IXE Q2W and the IXE Q4W arms than in the placebo arm (**Table 2**).

**Table 2:** Spine or pelvis MRI score changes in IL-17i trials in axial spondyloarthritis.

Study ID	Drug	Acronym	MRI endpoint	Arms	Number of patients with MRI	Baseline	Changes
NCT02696031	SEC	PREVENT	Berlin SIJ MRI score change	Placebo	139	2.70 (3.96)	-0.59
				SEC LD + 150mg Q4W	132	2.80 (3.83)	-2.38
				SEC NL + 150mg Q4L	134	2.24 (3.29)	-1.42
NCT02696798	IXE	COAST-V	SPARCC Spine Score change from baseline to W16	Placebo	87	15.8 (21.2)	-1.51 (1.15)
				ADA 40mg Q2W	90	20.0 (28.4)	-11.57 (1.11)
				IXE 80mg Q2W	83	16.6 (23.8)	-9.58 (1.17)
				IXE 80mg Q4W	81	14.5 (20.6)	-11.02 (1.16)
			SPARCC SIJ Score change from baseline to W16	Placebo	87	5.0 (9.6)	0.9 (0.6)
				ADA 40mg Q2W	90	4.7 (11.2)	-4.2 (0.6)
				IXE 80mg Q2W	83	6.4 (10.9)	-4.3 (0.6)
				IXE 80mg Q4W	81	4.5 (9.1)	-4.0 (0.6)
NCT02757352	IXE	COAST-W	SPARCC spine Score change from baseline to W16	Placebo	51	6.4 (10.2)	3.3 (1.4)
				IXE 80mg Q2W	58	11.1 (20.3)	-4.0 (1.5)
				IXE 80mg Q4W	53	8.3 (16)	-3.0 (1.4)
NCT02696785	IXE	COAST-X	SPARCC SIJ Score change from baseline to W16	Placebo	105	6.2 (9.1)	-0.31 (0.54)
				IXE 80mg Q2W	102	7.5 (10.8)	-4.52 (0.53)
				IXE 80mg Q4W	96	5.3 (8.3)	-3.38 (0.55)
NCT02963506	BKZ	BE AGILE	SPARCC SIJ Score change from baseline to W12	Placebo	20	NR	-3.7
				BKZ 16mg Q4W	20	NR	-10
				BKZ 64mg Q4W	20	NR	-9.5
				BKZ 160mg Q4W	20	NR	-2.5
				BKZ 320mg Q4W	20	NR	-5.5
			Berlin Spine MRI score change from baseline to W12	Placebo	20	NR	-1.3
				BKZ 16mg Q4W	20	NR	0.5
				BKZ 64mg Q4W	20	NR	-1.8
				BKZ 160mg Q4W	20	NR	-3.5
				BKZ 320mg Q4W	20	NR	-3.1

SEC, secukinumab; IXE, ixekizumab; BKZ, bimekizumab; SPARCC, Spondyloarthritis Research Consortium of Canada; SIJ, sacroiliac joint; NR, not reported.

Sustained efficacy was observed at Week 52 for both COAST-V and COAST-W trials (56). At Week 52, 47% - 53% of patients in the COAST-V trial and 31% - 39% patients in the COAST-W trial achieved an ASAS40 response and demonstrated sustained clinical efficacy of IXE. Moreover, patients originally randomized to placebo showed rapid improvement in ASAS40 response rates after switching to IXE and by 52 weeks responses were similar to those seen in patients originally randomized to IXE. In the COAST-V trial, patients originally randomized to Adalimumab demonstrated further improvement after switching to IXE with ASAS40 response of 36% at week 16 increasing to 51.2% at week 52. Treatment with IXE was well tolerated in this 52-week extension. Serious infections were reported by 3 patients in COAST-V and 3 patients in COAST-W but only 1 patient withdrew from the study. Candida infection was reported by 2 patients in each of the COAST-V and COAST-W studies. Two patients reported *de novo* Crohn’s in COAST-V and 2 reported flares of ulcerative colitis but there was no association between these events and length of exposure to IXE. There were no events related to colitis in COAST-W. Acute anterior uveitis (AAU) was reported by 6 patients (1.8%) in COAST-V and 11 (3.9%) in COAST-W of whom 14 had a history of AAU. Corresponding exposure adjusted incidence rates (IRs, number of events per 100 patient years) for Crohn’s disease and ulcerative colitis were 0.8 and 0.4, respectively, while the exposure adjusted IR for AAU was 3.9. These event rates are comparable to those reported previously for these extra-articular manifestations in patients receiving TNFi bDMARDs for AS (57, 58).

IXE has also demonstrated efficacy in a 52-week placebo-controlled trial of nr-axSpA (COAST-X) (59). Patients refractory to ≥2 NSAIDs with either elevated CRP or MRI inflammation in the SIJ and meeting ASAS classification criteria for axSpA were randomized 1:1:1 to IXE 80mg Q4W, Q2W or placebo. Escape to open label IXE Q2W was possible after week 16 at investigator discretion. Primary endpoint was the ASAS40 response at weeks 16 (European Union and non-US analysis) and week 52 (US analysis). It was achieved at 16 weeks by 35%, 40%, and 19% of patients in the IXE 80mg Q4W, IXE 80mg Q2W, and placebo groups, respectively (**Table 1**). At 52 weeks, the corresponding ASAS40 response rates were lower at 30%, 31%, and 13%. However, 25% on ixekizumab Q4W, 17% on ixekizumab Q2W, and 6% on placebo had ASAS40 at their last visit before switching. Statistically significant group differences were seen as soon as week 1 and all major secondary endpoints showed greater improvement in the IXE groups compared to those on placebo. These included the ASDAS, BASDAI, SF-36 Physical Component Scale, and SPARCC MRI SIJ inflammation score (**Table 2**). The frequencies of SAE, discontinuations due to adverse events, and frequency of anterior uveitis were similar across the three groups. A single case of Crohn’s disease occurred in a patient reported as having pre-existing diarrhea.

In a withdrawal study (COAST -Y), patients in the previous COAST clinical trial program who completed 52 weeks of treatment with IXE continued a further 24 weeks on IXE, and those who achieved ASDAS-ID were randomly assigned to continue IXE Q2W, Q4W or to receive placebo (60). The primary endpoint was the proportion of patients who had not flared, which was defined as an ASDAS≥2.1 at two consecutive visits or an ASDAS>3.5 at any visit, at the 40-week time point of the randomized withdrawal period, with time-to-flare as a major secondary endpoint. A total of 773 patients were enrolled and 155 were randomized to treatment withdrawal or continuation on treatment with IXE. At 40 weeks, 83.3% of those who continued treatment with IXE had not flared as compared to 54.7% of those on placebo, and IXE significantly delayed time to flare. Re-treatment of patients who flared with at least 16 weeks of open-label IXE resulted in recapture of ASDAS-ID in only 44% of those who had withdrawn to placebo.

Overall, the IXE RCT data demonstrated convincingly that IL-17A targeted therapy with this agent is effective at ameliorating inflammation in r-axSpA as well as nr-axSpA and in bDMARD naïve as well as TNFi experienced patients. It has a clearly beneficial impact on objective manifestations of disease, namely, the CRP and MRI inflammation in the SIJ and spine, but impact on concomitant musculoskeletal inflammation in the peripheral skeleton, such as enthesitis and peripheral synovitis, has not yet been reported in axSpA. An important consideration is that disease flare occurs upon treatment withdrawal in about half of patients over a 40-week time frame and re-institution of therapy recaptures the response in only half of the patients that flare.

### Bimekizumab

Bimekizumab (BKZ) is a monoclonal humanized IgG1 antibody that neutralizes both IL-17A and IL-17F. Its efficacy in active AS was investigated in a phase 2b placebo controlled, double blinded, dose ranging trial (61). The primary endpoint was the proportion of patients who achieved the ASAS40 at Week 12. Secondary endpoints included the ASAS20 response, change in the BASDAI, change in the BASFI, and change in the ASDAS at Week 12. A total of 303 patients were randomized 1:1:1:1:1 into placebo, BKZ 16mg, 64mg, 160mg, or 320mg Q4W. At Week 12, more BKZ-treated patients achieved an ASAS40 response compared to placebo (29.5% - 46.7% *vs* 13.3%, p < 0.05 in all comparison, **Table 1**). In addition, a significant dose-response was observed. In patients treated with BKZ, the ASAS20 response at Week 12 was achieved by 41% (BKZ 16mg Q4W) to 72.1% (BKZ 320mg Q4W) of patients, compared to 28.3% in the placebo arm. Efficacy was maintained at 48 weeks, and 58.6% patients in the BKZ 160mg Q4W arm and 62.3% patients in the BKZ 320mg Q4W arm achieved the ASAS40 response. During the 48-week study period, the most reported adverse event was nasopharyngitis, with 34 cases out of 303 patients. Candidiasis infection was reported in 19 patients, major cardiovascular events in 2 patients, and IBD in 4 patients, two being *de novo* events. No malignancy or suicidal ideation was reported. Thirteen patients had SAE, and 20 patients discontinued the study drug due to AEs. Some patients in the study had MRI spine at baseline and at Week 12. In addition to the symptom relief, inflammatory lesions seen on spinal and SIJ MRI were reduced after 12 weeks of BKZ (**Table 2**).

Overall, one can conclude that treatment with BKZ is efficacious and well-tolerated though it is questionable whether clinical and MRI improvement in disease activity is superior to responses observed with bDMARDs targeting only IL-17A. While different patient populations recruited to these clinical trials requires caution in making comparisons between outcomes in different clinical trials, it nevertheless appears unlikely that the dual inhibition hypothesis is supported by the results of the efficacy data related to clinical outcomes in this phase 2b trial of BKZ. An important future priority will be to determine whether this agent might prevent the development of ankylosis, which would support the dual inhibition hypothesis and be consistent with the *in-vitro* data that blockade of both IL-17A and IL-17F is more effective at preventing osteogenic differentiation of periosteal stem cells than blockade of either cytokine alone.

### Netakumab

Netakumab (NTK) is a recombinant humanized IgG1 IL-17A monoclonal antibody with modified CDR-regions and Fc-fragment. In a phase 2, double blinded RCT, patients with active AS were randomized 1:1:1:1 to placebo *vs*. NTK 40mg, 80mg, 120mg Q2W (62). The primary endpoint was the percentage of patients achieving the ASAS20 response at Week 16. The ASAS40 response at Week 16 was assessed as one of the secondary endpoints. A total of 88 patients were included in the study, with 22 patients in each arm. At Week 16, 72.7%, 81.8% and 90.9% patients in the NTK 40mg, 80mg, and 120mg arms achieved an ASAS20 response, compared to 42.8% patients in the placebo arm, and significantly more in the NTK 80mg and 120mg arms (**Table 1**). An ASAS40 response was achieved in 40.9%, 63.6% and 72.7% patients in the NTK 40mg, 80mg, and 120mg arms respectively, compared to 14.3% in the placebo arm. No SAE or withdrawal due to AE was observed during the 16-week study period. However, it was a relatively small study with a short study duration. More data is needed to demonstrate its efficacy and safety in treating patients with axSpA.

### Safety of IL-17 Inhibition

Although patients with monogenic diseases with loss of IL-17 have increased risk for candidiasis, monoclonal antibodies against IL-17A seem to be relatively safe. Using post-marketing surveillance data and pooled clinical trial data, exposure adjusted IR of adverse events of SEC in patients with AS per 100 patient-years was 1.2 for serious infections, 0.7 for candida infections, 0.6 for major adverse cardiac events, 0.4 for Crohn’s disease, 0.2 for ulcerative colitis, and 0.5 for malignancy (63). IXE reported a similar safety profile from pooled clinical trial data and showed that the adjusted IR per 100 patient-years was 1.3 for serious infections and infestations, 1.6 for candida infection, 0.1 for major adverse cardiac events, 0.5 for Crohn’s disease, 0.4 for ulcerative colitis, 0.4 for malignancy (64). No reactivation of tuberculosis was observed in these two studies, and demyelinating disease was not commented.

### Real-World Data

Consistent with the efficacy and safety data from clinical trials, in clinical practice, IL-17 inhibitor was shown to be effective. In patients with axSpA started on SEC as part of routine clinical care (N =1860), the drug retention rate at 12 months was reported to be 72%. Twenty two percent of patients reached ASAS40 and 26% had ASDAS major improvement (ASDAS-MI, ASDAS decrease ≥2.0). When drug retention was examined in TNFi naïve AS patients, the drug retention rate of SEC at 12 months was increased to 84%, and 55% of patients reached ASAS40 and 51% had ASDAS-MI at 12 months (65).

## Clinical Studies of IL-17 in Spondyloarthritis Related Diseases

Genetic and epidemiologic studies have established the association between AS and psoriasis, uveitis, and IBD. More than one third of patients with AS report one or more extra-articular manifestations, including psoriasis, uveitis and IBD (66). However, despite the shared genetic risk factors in the IL-23/IL-17 pathway, and a positive response to IL-23 inhibitors in psoriasis/PsA and IBD, the efficacy of IL-17i in SpA-related conditions varies.

Phase 3 clinical trials of SEC, IXE and BKZ have demonstrated clear efficacy of IL-17i in psoriasis and PsA. Among them, the MAXIMISE trial is a phase 3b, randomized, double blind trial that focuses on axial PsA, a condition that may be distinct from axSpA, but with overlapping features (67). In this study, patients with PsA fulfilling Classification criteria for Psoriatic Arthritis (CASPAR) criteria, with active spinal symptoms [defined as BASDAI ≥ 4 and spinal pain ≥ 40mm (0-100mm visual analog scale)], and inadequate response to NSAIDs were included. A total of 498 patients were randomized 1:1:1 into SEC 300mg Q4W *vs* SEC 150mg Q4W *vs*. placebo arms. Two-third of the patient population had Grade 1 to Grade 4 sacroiliitis on either side as determined by the local investigator, although the number meeting mNY criteria for radiographic sacroiliitis was not stipulated. Around 60% of patients had a positive MRI with inflammation in the spine and SIJs, although the criteria used to define this were not stipulated. Moreover, Berlin scores for spinal inflammation at baseline were less than half of those recorded in a trial of a TNFi agent (certolizumab) in patients with r-axSpA and even less than in those patients with nr-axSpA. HLA-B27 status was positive for 33% of the 261 patients for whom this data was available (68). The primary endpoint was the proportion of patients achieving ASAS20 response at Week 12, and secondary endpoints included the ASAS 40 response at Week 12. At Week 12, 63% patients in the SEC 300mg Q4W arm and 66% patients in the SEC 150mg Q4W arm achieved an ASAS20 response, compared to 31% in the placebo arm. ASAS40 response rates were 44%, 40% and 12% respectively. At Week 52, the retention rates were 83% for SEC 300mg Q4W arm and 86% for SEC 150mg Q4W arm. The study also included exploratory outcomes such as improvement of Berlin MRI score for spine and SIJ at Week 12. Although the baseline MRI scores for spine and SIJ were relatively low, the least square means of difference from baseline to Week 12 between SEC arms and the placebo arm were significant. However, the magnitude of response was substantially less than observed in an RCT of a TNFi in patients with r-axSpA and nr-axSpA (68). A significant drawback of this study is the lack of central evaluation of radiographs and lack of detail as to how MRI positivity for inflammation in the spine was defined. The age of the patients was higher than typically noted for AS trials so it is likely that many patients will have had MRI inflammation related to degenerative changes in the spine and SIJs. The 33% prevalence of HLA-B27 in this population was lower than the 60% previously reported in psoriatic axSpA further suggesting that some patients may have had mechanical causes of back pain and MRI inflammation. Moreover, 80% had concomitant peripheral arthritis and the self-reported improvement in clinical outcomes of pain and stiffness could reflect predominant alleviation of peripheral arthritis. However, the improvement in MRI inflammation in SEC-treated patients supports the hypothesis that IL-17A is a cytokine that also mediates inflammation in the axial joints of patients with PsA.

In contrast, the efficacy of IL-17 blockade in uveitis has been inconclusive and the most recent data indicates it is not effective. Subcutaneous loading and maintenance dosing of SEC were tested in three phase 3 randomized, double-blind trials for Behcet’s uveitis, active non-Behcet’s, noninfectious uveitis, and quiescent, non-infectious, non-Behcet’s uveitis, respectively, and none of the studies met its primary endpoint for efficacy (69). However, in a proof-of-concept phase 2 study with 37 patients, when given intravenously at 10mg/kg for loading and maintenance, SEC seemed effective compared to subcutaneous dosing and placebo (70). It is worth noting that the sample size of this study was very small, and all the uveitis trials only included patients with intermediate, posterior, and pan-uveitis, whereas in patients with AS, anterior uveitis is much more common. On the other hand, from the safety perspective, IL-17 blockade does not appear to trigger uveitis flares in patients with AS. In a pooled analysis of three phase 3 RCTs of SEC in AS, the exposure adjusted IR for uveitis was 1.4 per 100 patient-years. In comparison, the exposure adjusted IR of uveitis during TNFi treatment was 2.6 to 3.5 per 100 patient-years (71). Caution is warranted in comparing event rates across trials because event rate during a trial will depend on the numbers of patients recruited with a prior history of uveitis, especially in the year preceding entry into the trial. A recent study reporting AAU event rates in the Swedish Rheumatology Quality Register was based on 4851 treatment starts (456 secukinumab; 4395 any TNFi); the rate of AU-diagnoses per 100 patient-years was 6.8 (95% CI 5.2 to 8.7) for secukinumab (72). Among the TNFi, the rate varied from 2.9 (95% CI 2.1 to 3.7) for infliximab and 4.0 (95% CI 3.3 to 4.9) for adalimumab to 7.5 (95% CI 6.7 to 8.4) for etanercept. Sensitivity analyses confirmed the pattern of higher AU rates with secukinumab and etanercept *versus* monoclonal TNFi.

For Crohn’s disease, it is surprising that IL-17A blockade is not only ineffective, but may potentially worsen disease activity and/or cause serious side effects. A phase 2 study of SEC in patients with active Crohn’s disease was terminated prematurely because it met the prespecified futility criteria (73). Another phase 2 study, which evaluated AMG 827 (Brodalumab), an anti-IL-17 receptor antibody, did not demonstrate efficacy either (74). Instead, in both studies, a disproportionate number of patients in the IL-17 blockade arms experienced worsening of their Crohn’s disease compared to placebo. Furthermore, in the SEC study, more patients experienced adverse events in the SEC arm (74%, 29/39) than in the placebo arm (50%, 10/20), particularly infections.

It is possible that the greater disease severity, as defined by higher rates of prior bowel surgery and failure of TNFi therapy, in patients randomized to SEC were contributory factors. More likely, these results suggest a different role of IL-17A in IBD, specifically, protective rather than pathogenic. In mice, IL-17A or IL-17 receptor deficient T cells induce flare of colitis when transferred into RAG-1 deficient mice, and blocking IL-17A increases tissue damage and leads to enhanced inflammation (75–77). Furthermore, it has been shown that IL-17A is produced in an IL-23 independent fashion by resident γδ T cells in the intestinal lamina propria and these cells have an important role in maintaining the mucosal barrier (77). These findings in murine models probably explain the unsuccessful clinical trials of IL-17A inhibitors beyond trial design. Interestingly, IL-17F deficient mice, but not IL-17A deficient mice, were found to be resistant to chemically induced colitis and T cell induced colitis, with increased T_reg_ population in colon, commensal dysbiosis (increased *Clostridium* cluster XIVa, reduced *Prevotellaceae*) and reduced expression of some antimicrobial peptides (78). Antibody against IL-17F was effective in treating chemically induced colitis, while antibody to IL-17A was not. Whether sole IL-17F inhibition would be effective in IBD patients is unclear.

What is reassuring is that, clinically, in patients with psoriasis, PsA or AS treated with SEC or IXE, the incidence of IBD was low, and the risk was similar to patients without this exposure (79–81). A recent retrospective pooled analysis of 7355 patients and a cumulative exposure time of 16,226.9 patient-years described the incidence rates of IBD in patients enrolled in 21 clinical trials of SEC for the treatment of psoriasis, PsA, and AS (81). There were 41 cases of IBD. Forest plots illustrating the relative risk of Crohn’s and UC *versus* placebo did not show any increased risk from treatment with SEC. There was also no evidence of a dose-response relationship between SEC dose (150 mg *vs* 300 mg) and rates of reported IBD and the exposure adjusted IRs did not increase over time for each patient or each indication. In the post-marketing safety surveillance analysis, the cumulative reporting rate of IBD remained stable at approximately 0.20 reported events per 100 PY over a cumulative exposure of >96,000 patient years.

Further, a recent study examined the intestinal biopsy samples from axSpA patients who developed clinical Crohn’s disease after IL-17A inhibition, and found an expansion of IL-17E producing cells (82). Whether a similar increase, or even higher levels might be observed in those without colonic inflammation is unknown. In contrast, in classic Crohn’s disease patients, the expression of IL-17E were reduced, and the reduction correlated with endoscopic severity, suggesting IL-17E, similar to IL-17A, might be protective in patients with Crohn’s disease (83).

These differences, both at the clinical level, and at the translational level, suggest that the underlying mechanism of IL-17A inhibition induced colonic inflammation might be different from Crohn’s disease.

## Clinical Studies of IL-23 Inhibition in Axial Spondyloarthritis

### Risankizumab

A proof-of-concept dose-ranging placebo-controlled RCT evaluated risankizumab (BI 655066/ABBV-066), a humanized, IgG1 monoclonal antibody that selectively targets the p19 subunit of IL-23, in patients with definite AS refractory to ≥ 2 NSAIDs (84). Radiographic sacroiliitis was determined by local reader evaluation. The dosing range of risankizumab had previously demonstrated efficacy in RCTs of psoriasis, PsA, and Crohn’s disease (85–88). A total of 159 patients were randomized 1:1:1:1 to risankizumab (18 mg single dose, 90 mg or 180 mg at day 1 and weeks 8, 16 and 24) or placebo for 24 weeks with escape treatment to 180 mg risankizumab being available at 16 weeks for patients not achieving an ASAS20 response at 12 weeks. Primary endpoint was the ASAS40 response at 12 weeks and this was achieved by 25%, 21% and 15% of patients in the 18 mg, 90 mg and 180 mg risankizumab groups, respectively, which was not significantly different from the placebo group (18%). A total of 96 (60.4%) patients switched to the escape 180mg risankizumab treatment arm and prolongation of treatment for up to 40 weeks did not substantially improve ASAS40 response rates. No significant differences were noted for ASAS20, ASAS5/6, or ASAS partial remission responses. There was a dose-dependent reduction in the CRP level and greater reduction in the ASDAS-CRP for the 18mg and 180mg Risankizumab groups compared to placebo. However, a clinically relevant reduction in ASDAS-CRP ≥ 1.1 was not achieved with risankizumab and the reduction was primarily driven by the change in CRP. Greater though modest reduction in SPARCC MRI spine inflammation scores for the risankizumab 90mg and 180mg groups when compared to placebo was noted at week 12 in patients with a clinical response. But SPARCC MRI scores at 12 weeks were no different among groups who switched to escape treatment. There were no differences between treatment groups in levels of the IL-23/T_H_17 pathway biomarker β-defensin 2 or in several biomarkers of bone remodeling (dikkopf-1, sclerostin, BMP-7 and osteocalcin). Treatment was well tolerated with no significant differences in adverse events between treatment groups and no reports of severe infections.

### Ustekinumab

Three RCTs have evaluated ustekinumab, a human monoclonal antibody targeting the p40 subunit found in both IL-12 and IL-23, after an open label pilot study of 20 patients with AS (TOPAS) demonstrated improvement in clinical and MRI parameters of spinal inflammation at 24 weeks after receiving ustekinumab 90mg at 0, 4, and 16 weeks (89). This agent is approved for the treatment of psoriasis, PsA, and Crohn’s disease, and improvement in spinal symptoms was reported in a subgroup of patients with PsA considered to have axial inflammation by their physician. All three RCTs evaluated ustekinumab at 45mg or 90mg *versus* placebo at weeks 0, 4, 16 and randomized 1:1:1 to these groups (90). Two studies recruited patients with active r-axSpA. One of these studies included patients refractory to ≥2 NSAIDs but naïve to bDMARD therapy while the second study included patients refractory to a single TNFi. A third study recruited patients with nr-axSpA refractory to ≥2 NSAIDs and could have been exposed to no more than one TNFi. The primary endpoint was the ASAS40 response at week 24 for the r-axSpA trials and the ASAS20 response at week 24 for the nr-axSpA trial. Patients failing to achieve ≥10% improvement from baseline in both total back pain and morning stiffness measures at both week 12 and week 16 could escape at week 16 to either open label golimumab (patients with r-axSpA) or were re-randomized in a blinded manner to ustekinumab 45mg or 90mg (patients with nr-axSpA). The presence of radiographic sacroiliitis and MRI inflammation in the SIJ typical of axSpA was assessed by a single central reader. Major secondary endpoints included the BASDAI50 response, ASDAS-CRP inactive disease, and change from baseline in the BASFI. Study continuation for all 3 studies was based on the week 24 results in 346 patients with r-axSpA who were naïve to bDMARD. This showed that primary and secondary endpoints were not met and so all 3 studies were discontinued. In particular, there was no significant difference in the proportions of patients who achieved an ASAS40 response in the ustekinumab 45 mg (31%) and 90 mg (28%) groups when compared to the placebo group (28%). There were also no significant differences in MRI inflammation scores. Assessment of treatment responses in r-axSpA patients refractory to anti-TNF demonstrated higher ASAS40 responses in the ustekinumab 45mg (19%) and 90mg (27%) groups compared to the placebo group (12%) but early discontinuation of this RCT with only 44% of the planned sample size precluded valid statistical analysis and clinical conclusions. Moreover, separation from placebo was not consistent across endpoints and there was no benefit of ustekinumab in reducing CRP in this RCT. Frequencies of adverse events were comparable among treatment groups with no new safety signals. Of particular interest, neither T_H_17 cytokines (IL-17A, IL-17F, IL-22, and IL-23) nor T_H_1 cytokines (IFNγ and IL-12p70) were dysregulated at baseline in AS patients compared with healthy controls. Moreover, ustekinumab had only a minor impact on biomarkers at weeks 4 and 16 with only MMP3, serum amyloid A (SAA), and IL-8 being significantly decreased, and this change did not correlate with clinical response.

These RCTs conclusively demonstrate a lack of impact of targeting IL-23 for alleviation of inflammation in axSpA according to both patient self-reported outcomes, objective measures of inflammation, and biomarkers reflecting inflammation and tissue turnover. These RCTs also highlight the limitations of open label studies in a disease where the primary outcomes assessed in clinical trials are patient self-reported assessments of symptoms such as pain, stiffness, and global well-being. The open label study of ustekinumab did report an overall reduction of 30-40% in MRI scores for inflammation in the SIJ and spine after 24 weeks but this is substantially less than the 60-70% reductions noted in trials of TNFi agents. Moreover, it has been shown that fluctuation over a 3-month time frame in MRI inflammation in the SIJ beyond measurement error may be observed in about 20% of patients despite stable NSAID and/or DMARD intake (91). Consequently, it is very important to design a clinical trial program for novel therapeutics in axSpA that includes an appropriately powered phase II placebo-controlled RCT with MRI inflammation as an endpoint since demonstration of significant treatment group differences in MRI scores has been a consistent indicator of an efficacious therapeutic.

### Guselkumab

A recent report has added yet another twist to the potential therapeutic role of agents targeting IL-23 (92). The efficacy of guselkumab (GUS), a human monoclonal IgG1λ antibody that selectively binds to the p19 subunit of IL-23, was examined in patients with back pain in a post-hoc analysis of two phase III placebo-controlled RCTs that evaluated this agent in active peripheral PsA (93). In DISCOVER-1, 381 pts with active PsA (≥ 3 swollen joints, ≥ 3 tender joints; C-reactive protein ≥ 0.3mg/dL despite standard therapies) and in DISCOVER-2, 739 pts with active PsA (≥ 5 swollen joints, ≥ 5 tender joints, CRP ≥ 0.6mg/dL despite standard therapies) were randomized 1:1:1 to GUS 100mg Q4W, GUS 100mg Q8W (Wk0, Wk4, then Q8W), or placebo. This analysis included patients with sacroiliitis at baseline who had either documented imaging confirmation of sacroiliitis in the past or pelvic X-ray confirmation of sacroiliitis at screening (pooled data from DISCOVER-1&2) based on local investigators’ judgment of presence/absence of sacroiliitis. Central reading of imaging was not performed. Efficacy was assessed by BASDAI score, BASDAI50 response, spinal pain, ASDAS-ID, and ASDAS-MI. There were 312 patients with axial involvement and those in the GUS arms had significantly greater clinical responses with BASDAI50 improvement noted in 40.5% and 37.9% of the 100mg Q8W and 100mg Q4W dosing arms, respectively, as compared to 19.1% of those on placebo. A major challenge in the interpretation of this data is whether the clinical responses truly reflect improvement in axial *versus* peripheral inflammation as impact of treatment on objective features of inflammation, especially MRI of the SIJ and spine, was not reported. A second major challenge is the lack of a case definition for psoriatic axial involvement so that appropriate patients can be selected for clinical trials. Again, this will most likely require evidence of both active and structural lesions on MRI of the SIJ and spine.

**Figure 3:**
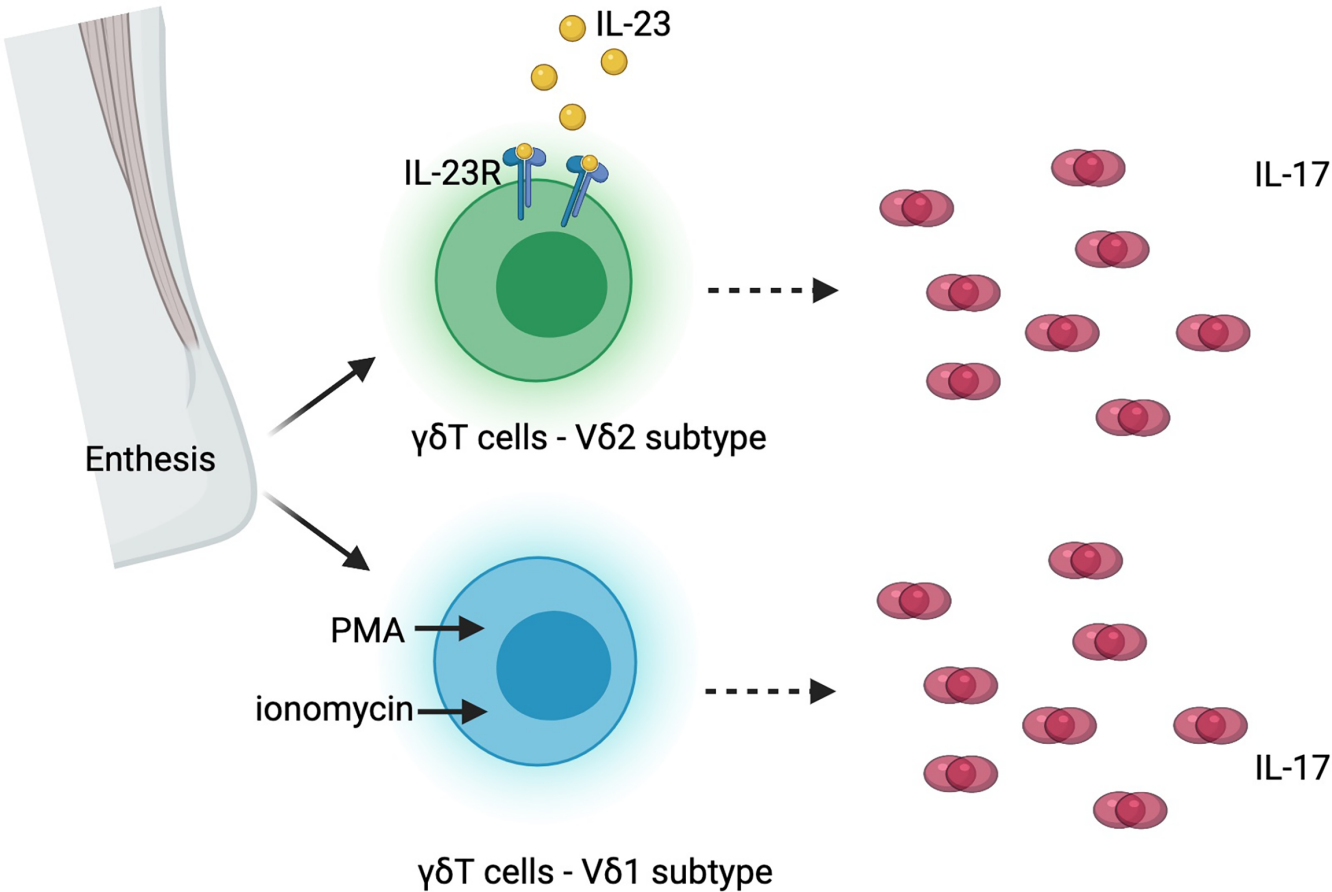
Co-existence of interleukin-23 (IL-23) dependent and independent interleukin-17 (IL-17) production in human enthesis. PMA, phorbol 12-myristate 13-acetate; IL-23R, interleukin-23 receptor.

Beyond the trial design issues outlined in the preceding paragraphs, the recent discovery of IL-17 production independent of IL-23 could be the explanation for the lack of success of IL-23 blockade in axSpA [ref]. Although IL-23 and IL-17 are closely related and form a classically described “IL-23/IL-17 pathway”, cells other than T_H_17 cells can be the source of IL-17, including MAIT cells, γδ T cells, NK cells and ILCs (**Figure 1**). These other RORγt+, Type 17 cells could express IL-17 upon stimulation by cytokines other than IL-23, and therefore, blocking IL-23/IL-23R would not be sufficient to suppress the downstream effects of IL-17. The co-existence of IL-23 dependent and independent IL-17 production has been supported by laboratory data both from human tissues and from animal models. In human entheseal tissue, two subsets of γδ T cells were identified, Vδ2 subset cells that express IL-23-inducible IL-17 associated transcripts, and Vδ1 subset cells that completely lack detectable IL-23R but express IL-17 transcript upon stimulation with phorbol 12-myristate 13-acetate (PMA) and ionomycin, suggesting the presence of IL-23 independent expression of IL-17 in human entheseal tissue (94) (**Figure 3**). Consistent with this finding and the importance of IL23-independent regulation of IL-17 in human disease, patients with AS have a higher frequency of IL-17A positive MAIT cells in peripheral blood and in synovial fluid (95). IL-7, but not IL-23, induces IL-17A expression in these MAIT cells (95). Inhibition of IL-23 in collagen-induced arthritis and SKG mice ameliorated inflammatory arthritis but did not completely abolish it (96, 97). Targeting IL-23R in the HLA-B27 transgenic rat, an experimental model that closely resembles human SpA, was not effective once arthritis was established but did have some beneficial impact in preventing the onset of disease, indicating IL-23 independent production of IL-17 in the perpetuation of disease (38). All these data suggest IL-17 can be induced by other cytokines in non-T_H_17 cells, and there could be a co-existence of IL-23 dependent and IL-23 independent IL-17 expression in entheseal tissues (**Figure 3**).

## IL-17 Effect on Bone Metabolism

Inflammation related bone loss and osteoporosis are often seen in patients with inflammatory arthritis, including patients with AS. Paradoxically, the hallmark of AS is new bone formation at the entheseal sites, particularly syndesmophyte formation and ankylosis of the spine. The mechanism of co-existence of new bone formation at entheseal sites and diffuse bone loss is not fully understood.

### IL-17 and Osteoclastogenesis

IL-17 has long been reported to play an important role in bone loss, by either inducing osteoclastogenesis or by suppressing Wnt signaling. Its effects likely depend on the type of cell and its stage of differentiation as well as the cellular composition and the extracellular milieu, especially the levels of other cytokines and inflammatory mediators. It may impact various cells mediating cartilage and bone destruction, but it may also act on mesenchymal cells and osteoblasts. Co-culture of T_H_17 cells and monocytes induced osteoclastogenesis in mice, and this process is possibly also mediated by IL-17 (98). In a coculture system of murine hematopoietic cells and primary osteoblasts, IL-17 induced expression of RANKL in osteoblasts, which in turn, induced differentiation of osteoclast progenitors into mature osteoclasts (99). In a mouse model of IL-17A mediated skin inflammation, keratinocytes, γδ T cells, and ILCs expressed IL-17A, and inhibited osteoblast and osteocyte function *via* inhibition of Wnt signaling (100). Consistent with this finding, more periosteal bone formation was observed at peak inflammation in IL-17A deficient mice (101). Furthermore, *in vitro* study showed that IL-17A inhibited osteoblast differentiation by inducing mRNA expression of the Wnt signaling antagonist secreted frizzled related protein-1 (sFRP1) and suppressing mRNA expression of sFRP3, which has been shown to promote osteoblast differentiation in bone marrow stromal cells (101). IL-17A might also have a direct effect on osteoclasts. In an *in vitro* study of human CD14+ monocytes, IL-17A upregulated RANK expression in osteoclast progenitors and therefore could directly induce osteoclastogenesis (102).

### IL-17 and Osteoblastogenesis

Other studies, which include murine fracture healing models and evaluation of human mesenchymal stem cells, suggest that IL-17 promotes osteoblastogenesis and new bone growth. In a murine fracture model, IL-17F was reported to induce osteoblast maturation and mediate the early phase of fracture repair (103). A further study in mice with bone injury demonstrated that IL-17A was produced immediately after injury by Vδ6+ γδ T cells in the peripheral tissues, and promoted osteoblastogenesis by increased proliferation of mesenchymal cells (104). In humans, activated T cells or derived condition medium triggers osteogenic differentiation in bone marrow derived mesenchymal stem cells, and this effect was mediated by IL-17A and IL-17F (105). In mesenchymal stem cells isolated from human spinal peri-entheseal bone, IL-17A enhances osteogenesis and suppresses adipogenesis (106).

The effect other of cytokines involved in IL-23/IL-17 pathway, in addition to IL-17, on bone formation and bone reabsorption has been reviewed elsewhere (107).

## Effect of IL-17 Inhibition in Patients With Ankylosing Spondylitis

In axSpA, preclinical data suggested that IL-17 may play a role in osteogenesis and that inhibition of IL-17 might be associated with less new bone formation. An *in vitro* study showed that IL-17A promotes osteogenesis in synovial fibroblasts from patients with active axSpA (39). Serum from patients with AS can induce osteogenesis in human periosteum-derived cells *in vitro*, and this effect can be blocked by bimekizumab, the dual inhibitor of IL-17A and IL-17F (19). Further, in the HLA-B27 transgenic rat model, administration of an IL-17A inhibitor antibody after disease onset resulted in new bone formation in 9 vertebrae from 3 rats, while treatment with control IgG2a resulted in new bone formation in 11 vertebrae from 5 rats, a numerical decrease but not statistically significant (39). It has been shown that circulating IL-17 increases with the onset of ankylosis in male DBA/1 mice, who develop enthesitis after being caged together, which then proceeds to ankylosis (108). Prophylactic administration of anti-IL-17 antibodies significantly prevented the development of ankylosis while administration after disease onset ameliorated but did not completely prevent ankylosis.

Clinical data for radiographic progression in patients with AS treated with IL-17A inhibitors is scant and inconclusive. Recent studies have used the modified Stoke AS Spine Score (50) (mSASSS, see **Box 1**) to quantify new syndesmophytes and radiographic progression in AS. In patients treated with SEC 150mg Q4W (n=86) *versus* 75mg Q4W (n=82) for 2 years, a comparable increase in mSASSS (0.30 +/- 1.94 in the 150mg dose group *vs*. 0.31 +/- 3.04 in the 75mg dose group) was observed (109). After 4-year treatment, the mean change in mSASSS was 1.2 +/- 3.91 in the 150mg dose group (N = 71) *vs*. 1.6 +/- 5.67 in the 75mg/up titration dose group (up titrate to 150mg, N = 23) *vs*. 1.8 +/- 4.32 75mg dose group (N = 61) (110), without a significant dose effect. When compared to a historical cohort of biologic naïve patients treated only with NSAIDs over 2 years, the change in mSASSS in SEC treated patients was 0.55 +/- 0.139 (N = 168), numerically lower than the change of mSASSS in the historical cohort (0.89 +/- 0.216, N = 69) (111). The proportion of patients without radiographic progression was also higher in the SEC group than historical cohort, but again, not statistically significant (mSASSS change <= 0: 60.7% in SEC group *versus* 52.2% in historical cohort; mSASSS change <= 2: 82.1.7% in SEC group *versus* 72.5% in historical cohort) (111).

However, the interpretation of these clinical data is limited by the reliability and sensitivity to change of the mSASSS and the absence of a control group which leads to a conservative bias to scoring change. This radiographic scoring method records radiographic features (sclerosis, squaring, erosion, syndesmophyte, ankylosis) in the anterior vertebral corners on lateral projections of cervical and lumbar spine radiographs, with a total possible score of 0-72 (50). In biologic naïve patients, 30 - 40% demonstrate an increase in mSASSS over 2 years, with an average change of 1.0 +/- 2.9 units (112). When used in clinical trials to assess the efficacy of a given treatment over placebo, a sample size of 100 in each arm would be needed to detect a difference between arms over 2 years (113). In addition, the inter-reader reliability was only 54% when assessing progression over 2 years (112). Consequently, the limited sample size in the above studies and measurement error preclude the demonstration of a statistically significant result for IL-17 inhibition on radiographic progression. Evidence of a lag effect of TNFi therapies on radiographic progression strongly suggests that at least 4-year follow up will be desirable which will necessitate active comparator studies as placebo-controlled trials will not be feasible. As yet, there are no soluble biomarkers that reflect radiographic progression which can be targeted in clinical trials but increasing evidence suggests that MRI inflammation in the spine may be a valid and more responsive surrogate.

## Conclusions

It has been a long way from the discovery of IL-23/IL-17 pathway to its clinical application in the treatment of patients with axSpA and SpA related conditions. While the inhibition of IL-17 cytokines has been a great success in controlling inflammatory symptoms in patients with axSpA, IL-23 blockade was not effective in treating axSpA. Despite that IL-17 inhibition is not effective and even harmful in treating IBD, but it does not seem to increase the incidence of IBD when given to patients with axSpA. It also seems ineffective for uveitis. These findings at the bedside brought new insights at the bench, suggesting tissue heterogeneity of expression and function of IL-23 and IL-17 cytokines. In addition to the classically described IL-23/IL-17 pathway, cells other than T_H_17 cells could express IL-17 in an IL-23 independent fashion. Laboratory evidence has suggested a co-existence of IL-23 dependent and independent IL-17 production in human entheseal tissues. Future work should focus on how to dissect these two processes at a clinical level in patients with axSpA, using objective measures for inflammation, such as CRP, MRI, and innovative biomarkers. Whether IL-17 inhibition will impede radiographic progression in patients with axSpA remains inconclusive. Future work should focus on improving study design, outcome measures, and identifying imaging and soluble biomarkers for disease progression.

## Author Contributions

RW and WM contributed to conception of the review, and conducted literature review. RW and WM wrote sections of the manuscript. All authors contributed to the article and approved the submitted version.

## Conflict of Interest

RW is the Founder of Garden State Rheumatology Consultants. WM is Chief Medical Officer of CARE Arthritis, has received research grants and consultancy fees from AbbVie, Novartis and Pfizer; and consultancy fees from Boehringer Ingelheim, Celgene, Galapagos, Gilead, Janssen, Lilly, Novartis, Pfizer and UCB Pharma.

## Publisher’s Note

All claims expressed in this article are solely those of the authors and do not necessarily represent those of their affiliated organizations, or those of the publisher, the editors and the reviewers. Any product that may be evaluated in this article, or claim that may be made by its manufacturer, is not guaranteed or endorsed by the publisher.
